# Supplementary material for: The sifting of visual information in the superior colliculus
Source: eLife. 2020 Apr 14;9:e50678. doi: 10.7554/eLife.50678 (PMC7237212; doi:10.7554/eLife.50678)
Supplement: Transparent reporting form [file elife-50678-transrepform.pdf]

1st Floor
24 Hills Road
Cambridge CB2 1JP, UK

P 01223 855340
W elifesciences.org
T @elife

eLife's transparent reporting form

We encourage authors to provide detailed information *within their submission* to facilitate the interpretation and replication of experiments. Authors can upload supporting documentation to indicate the use of appropriate reporting guidelines for health-related research (see EQUATOR Network), life science research (see the BioSharing Information Resource), or the ARRIVE guidelines for reporting work involving animal research. Where applicable, authors should refer to any relevant reporting standards documents in this form.

If you have any questions, please consult our Journal Policies and/or contact us: editorial@elifesciences.org.

Sample-size estimation

- You should state whether an appropriate sample size was computed when the study was being designed
- You should state the statistical method of sample size computation and any required assumptions
- If no explicit power analysis was used, you should describe how you decided what sample (replicate) size (number) to use

Please outline where this information can be found within the submission (e.g., sections or figure legends), or explain why this information doesn't apply to your submission:

We did not conduct a power analysis ahead of time, because the study does not test a specific binary hypothesis. We do measure and describe the graded differences between visual response properties of neurons in the superficial and deep layers of mouse superior colliculus (SC). For example, we noted that neurons with high looming selectivity are concentrated in the deep SC. These observations were made in early exploratory experiments and subsequently replicated in another round of recordings. The manuscript analyzes data from only these validation experiments. The effects reported rise far above any threshold for statistical significance.

Replicates

- You should report how often each experiment was performed
- You should include a definition of biological versus technical replication
- The data obtained should be provided and sufficient information should be provided to indicate the number of independent biological and/or technical replicates
- If you encountered any outliers, you should describe how these were handled
- Criteria for exclusion/inclusion of data should be clearly stated
- High-throughput sequence data should be uploaded before submission, with a private link for reviewers provided (these are available from both GEO and ArrayExpress)

Please outline where this information can be found within the submission (e.g., sections or figure legends), or explain why this information doesn't apply to your submission:

We report how many neurons were included in each analysis and how many recordings they came from in the figures themselves (Fig 2C, 3B, 3C, 4B, 4D). We also describe in detail how these neurons were selected in the Methods section.

eLife Sciences Publications, Ltd is a limited liability non-profit non-stock corporation incorporated in the State of Delaware, USA, with company number 5030732, and is registered in the UK with company number FC030576 and branch number BR015634 at the address 1st Floor, 24 Hills Road, Cambridge CB2 1JP | August 2014

Statistical reporting

- Statistical analysis methods should be described and justified
- Raw data should be presented in figures whenever informative to do so (typically when N per group is less than 10)
- For each experiment, you should identify the statistical tests used, exact values of N, definitions of center, methods of multiple test correction, and dispersion and precision measures (e.g., mean, median, SD, SEM, confidence intervals; and, for the major substantive results, a measure of effect size (e.g., Pearson's r, Cohen's d)
- Report exact p-values wherever possible alongside the summary statistics and 95% confidence intervals. These should be reported for all key questions and not only when the p-value is less than 0.05.

Please outline where this information can be found within the submission (e.g., sections or figure legends), or explain why this information doesn't apply to your submission:

We use a statistical method to decide whether a neuron is visually responsive based on its response to a single trial of the stimulus. The details can be found in the Methods section.

The Methods section and the figure legends also describe the Bonferroni correction for multiple comparison and define the dispersion and precision measures (Figs 4D and 5).

In Figs 2C, 3B, 3C, and 4B, we test the differences between the two empirical distributions (superficial vs. deep SC neurons) with the two-sample Kolmogorov-Smirnov test and report the *p*-values in the figure panels.

(For large datasets, or papers with a very large number of statistical tests, you may upload a single table file with tests, Ns, etc., with reference to sections in the manuscript.)

Group allocation

- Indicate how samples were allocated into experimental groups (in the case of clinical studies, please specify allocation to treatment method); if randomization was used, please also state if restricted randomization was applied
- Indicate if masking was used during group allocation, data collection and/or data analysis

Please outline where this information can be found within the submission (e.g., sections or figure legends), or explain why this information doesn't apply to your submission:

The Methods section and Figure 1 – Figure supplement 1 describe how the neurons recorded from multiple recordings were allocated to either the superficial or the deep SC population.

Additional data files ("source data")

- We encourage you to upload relevant additional data files, such as numerical data that are represented as a graph in a figure, or as a summary table
- Where provided, these should be in the most useful format, and they can be uploaded as "Source data" files linked to a main figure or table
- Include model definition files including the full list of parameters used
- Include code used for data analysis (e.g., R, MatLab)
- Avoid stating that data files are "available upon request"

Please indicate the figures or tables for which source data files have been provided:

We list the parameters used in the model of Fig 6 in Table 1. We have also attached a code used for analyzing the stimulus selectivity of recorded neurons (Fig 2). We will provide the source data once the paper is accepted.

eLife Sciences Publications, Ltd is a limited liability non-profit non-stock corporation incorporated in the State of Delaware, USA, with company number 5030732, and is registered in the UK with company number FC030576 and branch number BR015634 at the address 1st Floor, 24 Hills Road, Cambridge CB2 1JP | August 2014
